# Supplementary figures and images for: Fatty acid binding protein 1 and fatty acid synthetase over-expression have differential effects on collagen III synthesis and cross-linking in Zongdihua pig primary adipocytes
Source: PLoS One. 2023 May 4;18(5):e0270614. doi: 10.1371/journal.pone.0270614 (PMC10159151; doi:10.1371/journal.pone.0270614)

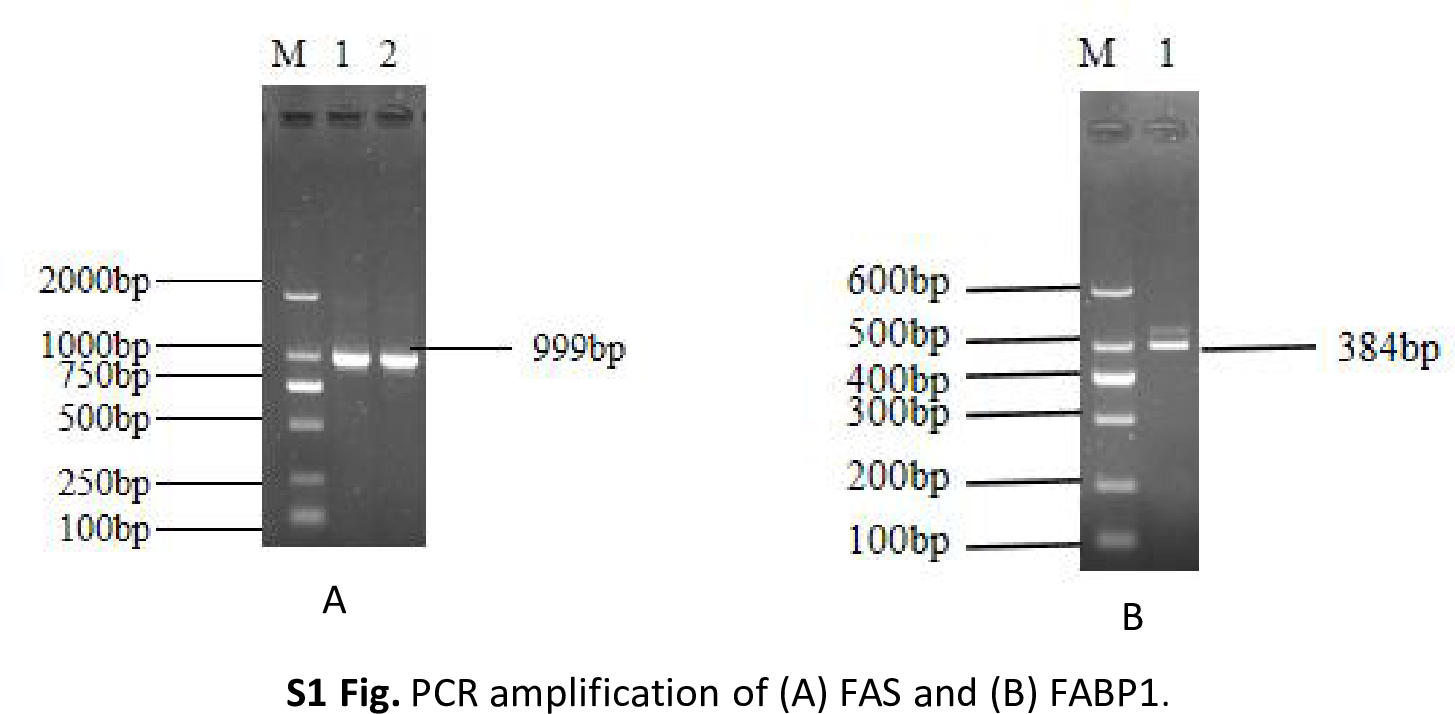

Supplement: S1 Fig — PCR amplification of (A) FAS and (B) FABP1. (TIF) [file pone.0270614.s001.tif]

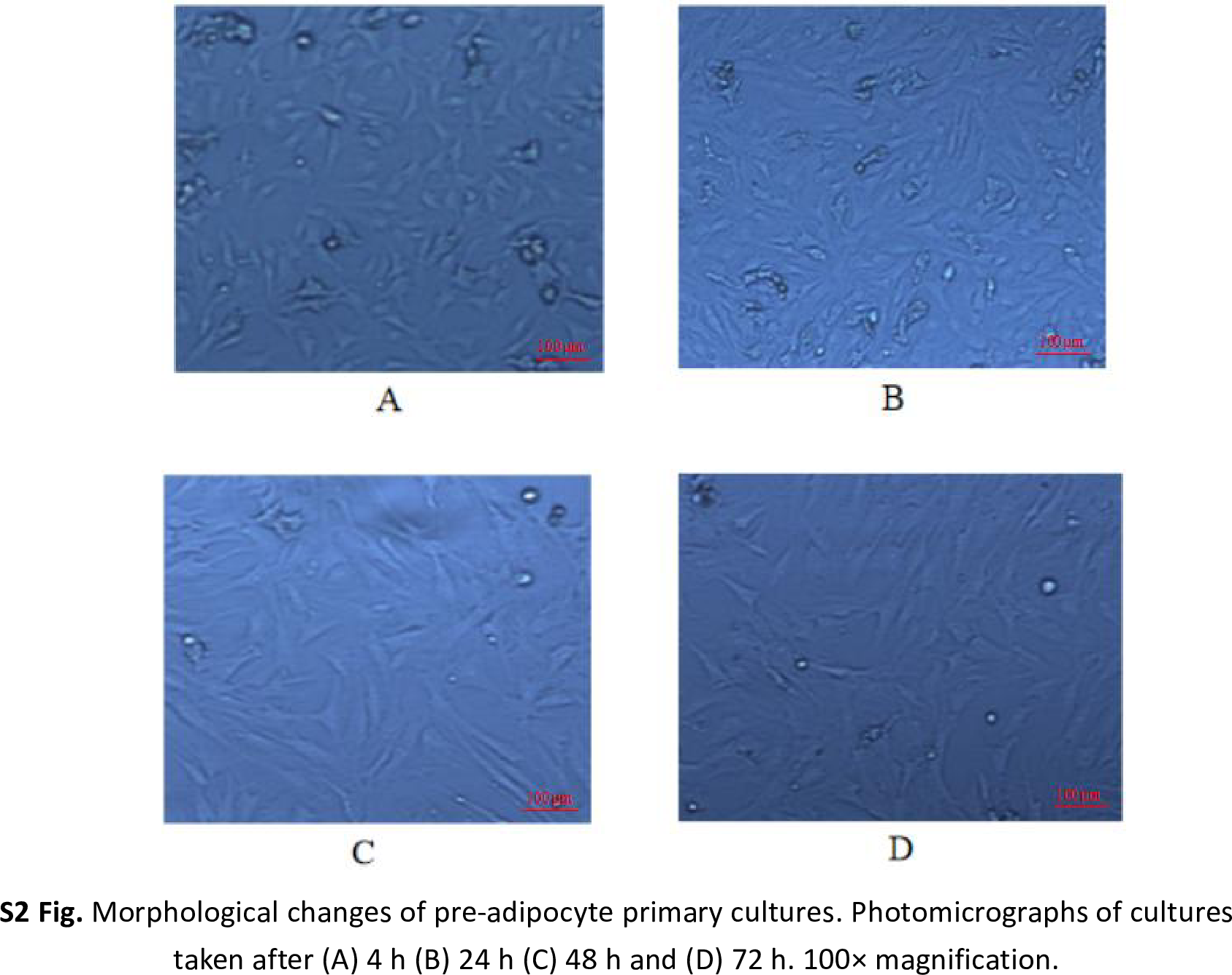

Supplement: S2 Fig — Photomicrographs of cultures taken after (A) 4 h (B) 24 h (C) 48 h and (D) 72 h. 100× magnification. (TIF) [file pone.0270614.s002.tif]

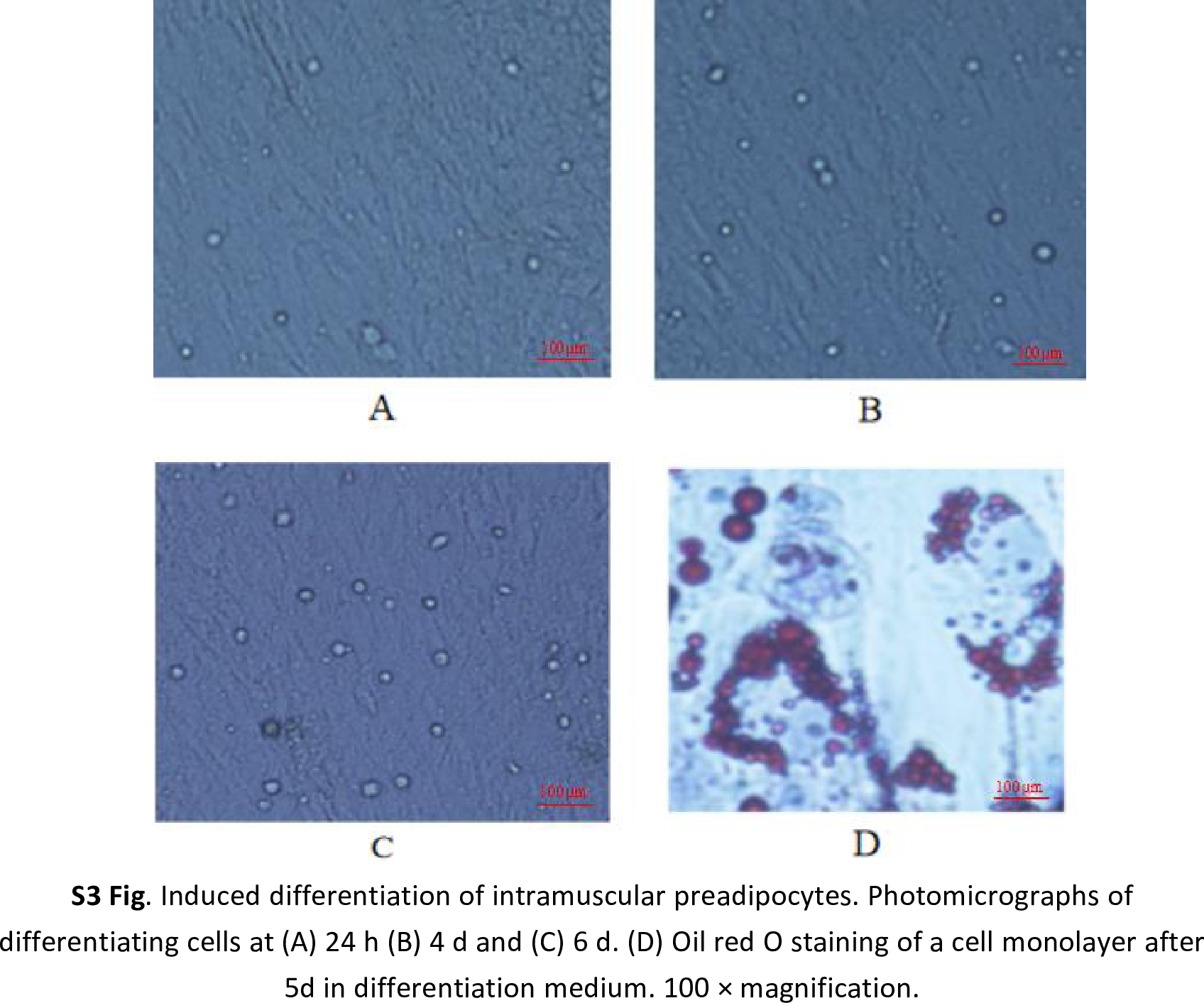

Supplement: S3 Fig — Photomicrographs of differentiating cells at (A) 24 h (B) 4 d and (C) 6 d. (D) Oil red O staining of a cell monolayer after 5d in differentiation medium. 100 × magnification. (TIF) [file pone.0270614.s003.tif]

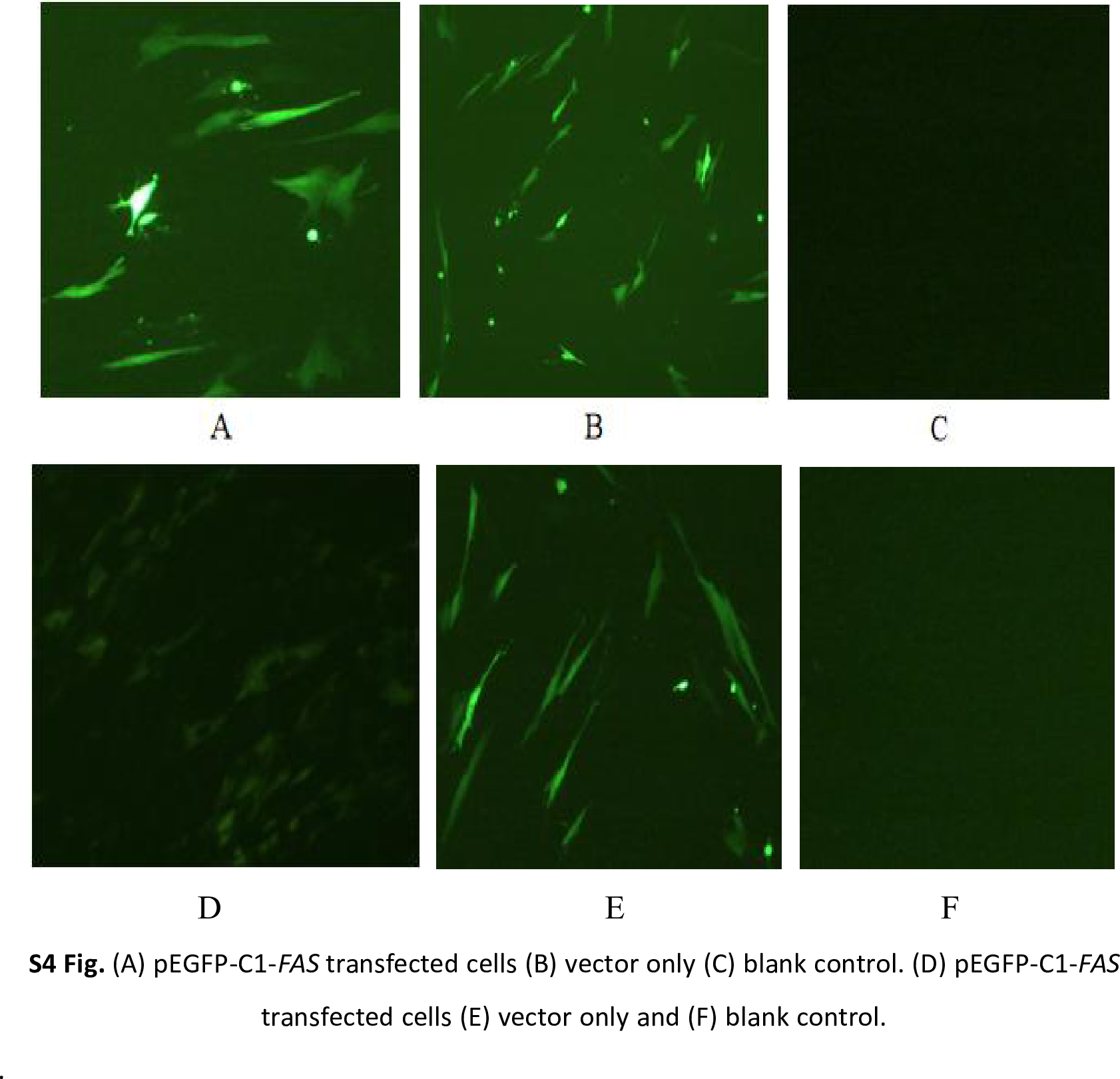

Supplement: S4 Fig — (A) pEGFP-C1-FAS transfected cells (B) vector only (C) blank control. (D) pEGFP-C1-FAS transfected cells (E) vector only and (F) blank control. (TIF) [file pone.0270614.s004.tif]

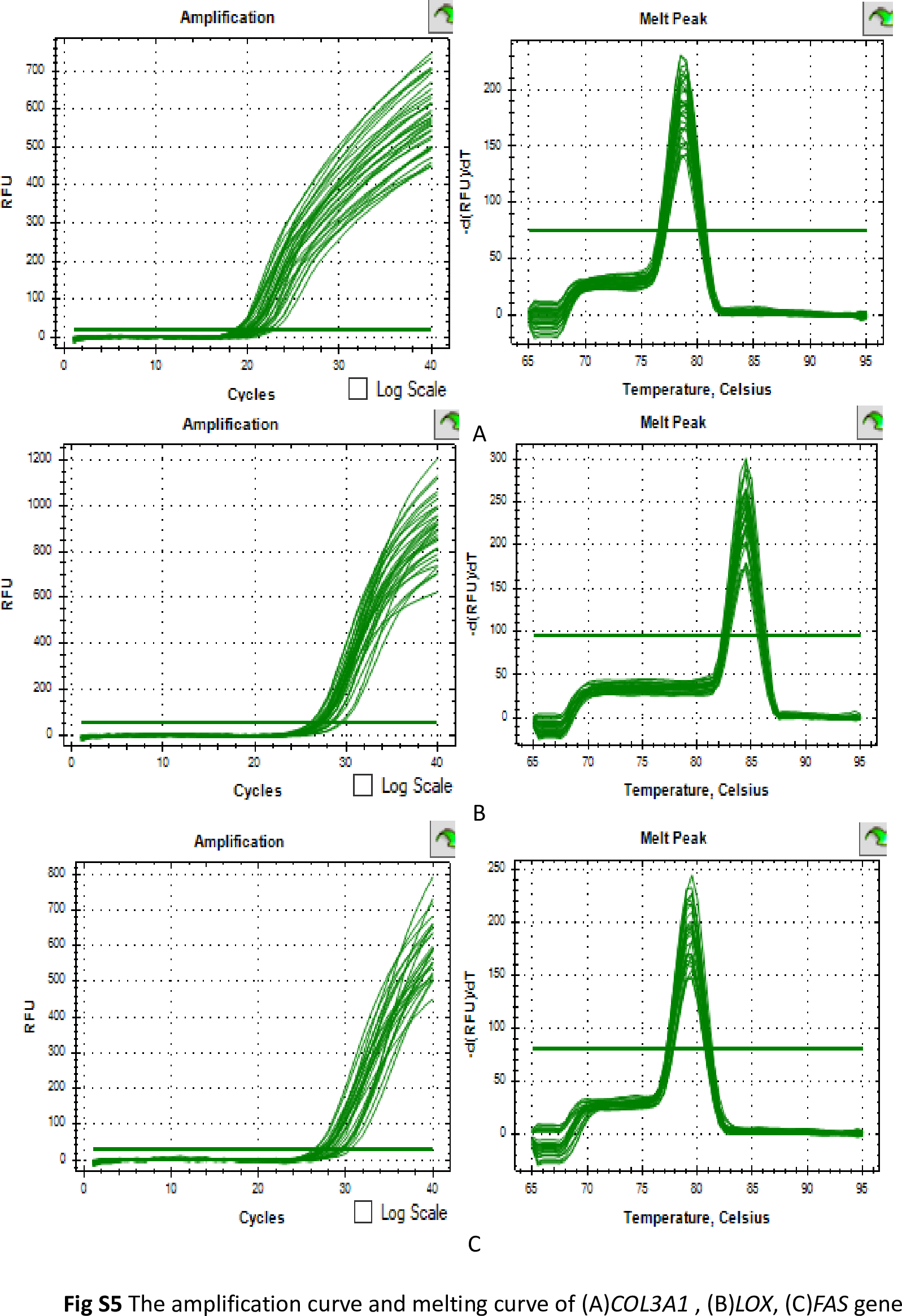

Supplement: S5 Fig — The amplification curve and melting curve of (A)COL3A1, (B)LOX, (C)FAS gene. (TIF) [file pone.0270614.s005.tif]

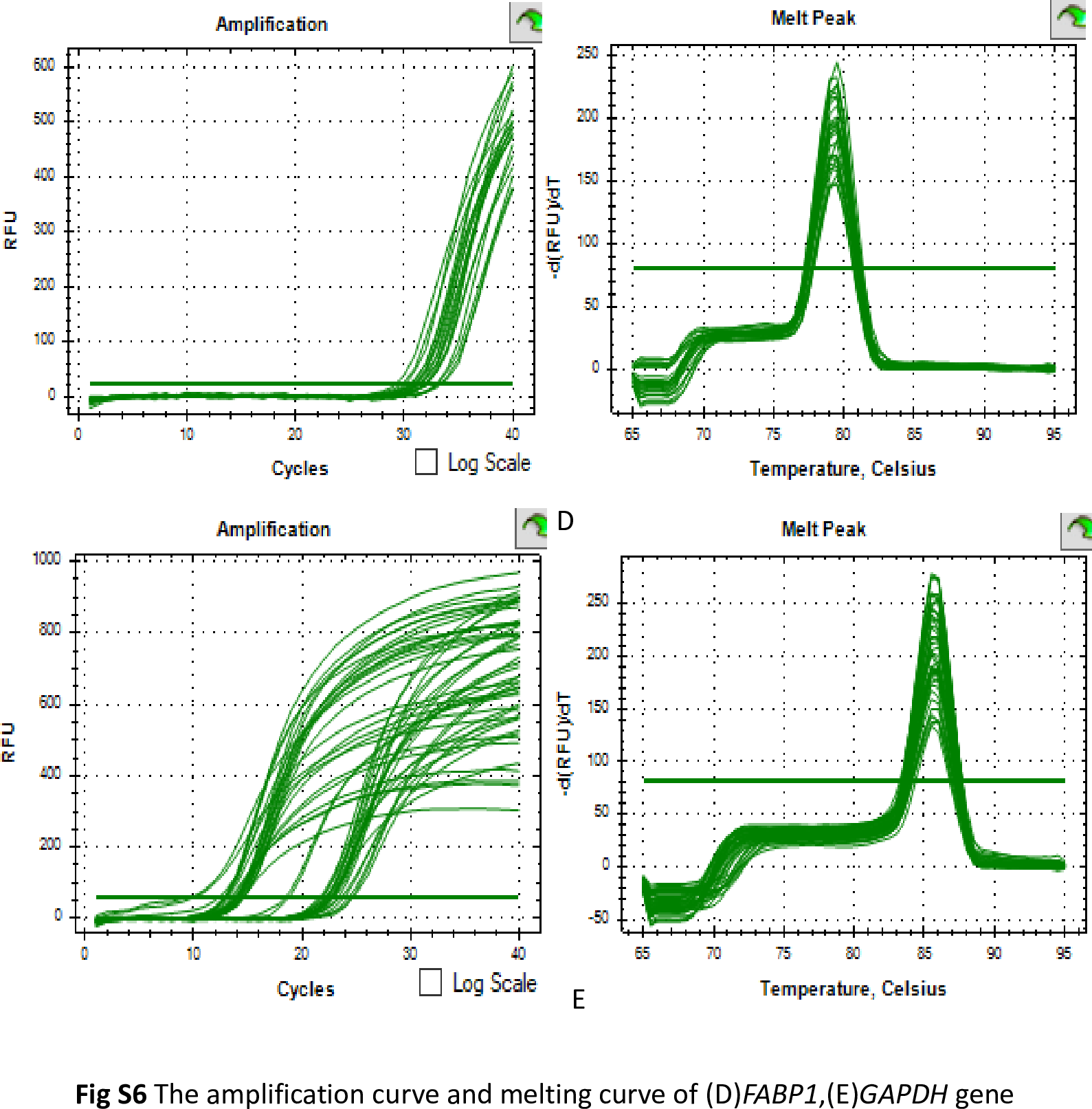

Supplement: S6 Fig — The amplification curve and melting curve of (D)FABP1, (E)GAPDH gene. (TIF) [file pone.0270614.s006.tif]
