# Supplementary material for: Fatty acid binding protein 1 and fatty acid synthetase over-expression have differential effects on collagen III synthesis and cross-linking in Zongdihua pig primary adipocytes
Source: PLoS One. 2023 May 4;18(5):e0270614. doi: 10.1371/journal.pone.0270614 (PMC10159151; doi:10.1371/journal.pone.0270614)
Supplement: Attachment — Submitted filename: Authors_review_05_2022.pdf [file pone.0270614.s007.pdf]

Dear authors,

The manuscript entitled "Fatty acid binding protein 1 and fatty acid synthetase overexpression have differential effects on collagen III and cross-linking in Zongdihua pig tissues" aims to determine whether FABP1 and FAS regulate expression of collagen and its crosslinking via lysyl oxidase in Zongdihua pigs. The manuscript points out that enhancing the expression of FAS and FABP1 increases collagen accumulation and this preliminarily suggests that FAS and FABP1 can serve as fat-related candidate genes providing a theoretical basis for the study of fat deposition in Zongdihua pigs. The manuscript brings interesting results, however, to be accepted for publication, it needs major changes and corrections. Below are suggestions to make the manuscript suitable for publication and more informative to the reader.

General suggestions

- Write all gene names in italics. Example: *FABP1*
- Please review the text for language.
- Some words are used in different ways in the manuscript such as cross-linking/crosslinking, Over-expression/Overexpression…Correct this, please.
- The introduction needs to be rewritten, addressing the issues related to the manuscript more deeply. In addition, give a clear hypothesis and objective in the introduction section.
- Please, change "longissimus dorsi" to "*Longissimus dorsi*" throughout the manuscript.
- Discussion needs to be rewritten, addressing more deeply the observed results.

Abstract

- Line 26 - Remove the **with** in the sentence "FAS encodes 333 amino acid hydrophobic protein containing with 26 phosphorylation sites and 0 transmembrane regions".
- Line 27 - Change the following sentence "The basal levels of FABP1 and FAS in pig tissues expression were 3 -3.5-fold higher in subcutaneous fat compared with muscle (P < 0.01)" to "The basal levels of FABP1 and FAS expression in pig tissues were 3 -3.5-fold higher in subcutaneous fat compared with muscle (P < 0.01)".
- Line 33- I believe you forgot to add "*FAS*" in this sentence "Therefore, enhancing FABP1 expression increases collagen accumulation...". Am I correct? Because the

overexpression of *FAS* and *FABP1* resulted in a significantly increased expression of the *COL3A1* gene.

- Keywords must be arranged in alphabetical order. It is not recommended to add words that are already in the title of the manuscript such as Zongdihua pig.

Introduction

- Add the title “Introduction” before the text in the line 40.
- You could improve your introduction by talking more about the breed (Zongdihua pig), mainly about the meat traits. It could also present some preliminary studies that address the evaluated genes deeply. All these issues need to be better integrated. In addition, the objective of the work should be better presented in this introduction.

Materials and methods

- Line 58 - Correct to Ethics Statements.
- Why is the topic "Ethics Statements" highlighted in the text?
- Please, I ask you to correct the text in relation to the language used. Some sentences are poorly worded.
- You don't write about the design and accession number of the following genes: *LOX* and *COL3A1* in the text.
- Did you work with the fold change (comparative CT method)? Why are the graphs with the Y-axis legend as a relative expression? I suggest reading the article "Analyzing real-time PCR data by the comparative CT method - Thomas D Schmittgen1 & Kenneth J Livak 2008" to correct this question.
- Subcutaneous fat and *Longissimus dorsi* samples were collected from how many animals? What is the N of this gene expression analysis? You need to better describe the experimental design of the study.

Results

- Why in the legend of figure 1 you used fat deposition if there is only information about the fold change between subcutaneous fat and *Longissimus dorsi*?
- Line 163:164 – “The expression levels for all 4 genes were extremely significantly ($P < 0.01$) elevated in subcutaneous fat tissues compared with the muscle (control) tissues (Figure 1).” Why did you compare subcutaneous fat to muscle? Your hypothesis should be very clear in the manuscript in relation to the comparisons made. Besides that, could you explain a little more about what you considered as a control in this study?

- Is the Y-axis legend correct in Figure 9?

Discussion

- Line 225:226 – Change the sentence "In this study, we found that FABP1 and FAS gene expression were significantly higher than for the control muscle tissue" to "In this study, we found that FABP1 and FAS gene expression were significantly higher in **subcutaneous fat** than for the control muscle tissue"
- Line 232 – Alter LOC gene to LOX.
- Your discussion of the results is superficial, you need to go deeper into the effect of the *FAS* and *FABP1* genes on the expression of *COL3A1* and *LOX* and the production of collagen. In addition to the effects of these genes on the deposition of fat and on meat traits.
- Line 231:233- "Our results agreed with previous studies indicating that *COL3A1* and *LOC* gene expression in subcutaneous fat are significantly higher and lower than in longissimus dorsi muscle tissues, respectively." - Figure 1 shows greater expression of both the *COL3A1* and *LOX* genes in subcutaneous fat in relation to *Longissimus dorsi*.
- You have not discussed deeply the effects of the expression of these genes on meat quality in the breed studied.
