# Supplementary material for: Fatty acid binding protein 1 and fatty acid synthetase over-expression have differential effects on collagen III synthesis and cross-linking in Zongdihua pig primary adipocytes
Source: PLoS One. 2023 May 4;18(5):e0270614. doi: 10.1371/journal.pone.0270614 (PMC10159151; doi:10.1371/journal.pone.0270614)
Supplement: Attachment — Submitted filename: Responses to editor.docx [file pone.0270614.s008.docx]

**Financial Disclosure Statement and Responses to Reviewers’ comments**

**II. Financial Disclosure Statement:**

This study was supported in part by two funding sources: 1) A special project for central guided local development funded from the Science and Technology Department of Guizhou Province to Dr. D. Zhou (Project No.: Qianke Central Land [2018]4015). Co-participants including R. Yang, Z. Zhao, Y. Wang, J. Li, L. Ren and, L. Xie. 2) A joint research project of local poultry industry in Guizhou province funded by Guizhou Provincial Department of Finance to Dr. D. Zhou (Project Number: Qian Cai Nong [2020] No. 175).

**II. Responses to Reviewers’ comments**

**Comments to the Author**

1. Is the manuscript technically sound, and do the data support the conclusions?

The manuscript must describe a technically sound piece of scientific research with data that supports the conclusions. Experiments must have been conducted rigorously, with appropriate controls, replication, and sample sizes. The conclusions must be drawn appropriately based on the data presented.

Reviewer #1: Partly

Reviewer #2: Yes

Reviewer #3: Partly

**Responses from the authors**: *We acknowledge the comments. Thanks!*

2. Has the statistical analysis been performed appropriately and rigorously?

Reviewer #1: I Don't Know

Reviewer #2: I Don't Know

Reviewer #3: No

**Responses from the authors**: *Acknowledged.*

3. Have the authors made all data underlying the findings in their manuscript fully available?

The [PLOS Data policy](http://www.plosone.org/static/policies.action#sharing) requires authors to make all data underlying the findings described in their manuscript fully available without restriction, with rare exception (please refer to the Data Availability Statement in the manuscript PDF file). The data should be provided as part of the manuscript or its supporting information, or deposited to a public repository. For example, in addition to summary statistics, the data points behind means, medians and variance measures should be available. If there are restrictions on publicly sharing data—e.g. participant privacy or use of data from a third party—those must be specified.

Reviewer #1: Yes

Reviewer #2: Yes

Reviewer #3: Yes

**Responses from the authors**: *Thanks!*

4. Is the manuscript presented in an intelligible fashion and written in standard English?

PLOS ONE does not copyedit accepted manuscripts, so the language in submitted articles must be clear, correct, and unambiguous. Any typographical or grammatical errors should be corrected at revision, so please note any specific errors here.

Reviewer #1: Yes

Reviewer #2: Yes

Reviewer #3: No

**Responses from the authors**: *We have our revised manuscript been proofread by a native English-speaking editor from a professional editing service.*

**Reviewers’ Comments to the Author**

Please use the space provided to explain your answers to the questions above. You may also include additional comments for the author, including concerns about dual publication, research ethics, or publication ethics. (Please upload your review as an attachment if it exceeds 20,000 characters)

**Reviewer #1:**

Title: Fatty acid binding protein 1 and fatty acid synthetase overexpression have differential
effects on collagen III and cross-linking in Zongdihua pig tissues

The current manuscript reports the initial basis on how the overexpression of FABP1 and FAS influence fat accumulation in adipocytes and in the expression of COL3A1 and LOX. The results may serve to other more complexes studies, and these future perspectives should have been reported. Moreover, an additional attention should be given to the manuscript before submission to avoid typos and the inclusion of incorrect references. In summary, my concerns are a) It was not reported the number of animals used for tissue extraction; b) the lack of protein abundance analysis to evaluate the functionality of the evaluated genes (mainly FAS, FABP1, COL3A1); c) the lack of references supporting the presence of FABP isoform 1 in muscle and adipocytes. Please consider the comments below.

**Responses from the authors**: *Thanks for your excellent summary comments. Your points are well taken. Per your suggestion, in the revised manuscript, we added some future perspectives in the Discussions/Conclusions sections. The revised manuscript has been proofread by a native English speaker to avoid typos and grammar misuses.*

*Regarding your concerns, a) we have added the number of animals used for tissue extraction explements; b) regarding protein abundance analysis, we highly agree with your suggestion. It was considered at the time; however, it was in January of 2020, the COVID-19 outbreak occurred in China. Still, the COVID control policy makes it difficult to conduct experiments. We will keep this in mind in our follow-up studies in the future; and c) thanks for the comment. We have added the following in the Introduction section with new references to support the presence of FABP isoform 1 in muscle and adipocytes. It reads in the revised manuscript (the actual numbering of the references will be adjusted in the manuscript):*

“FABP1 and FAPB2 genes play an important role in the regulation of fat metabolism, and FABP1 gene is expressed in various tissues of pigs [1]. Previous study showed that duck L-FABP or FAPB1 gene polymorphism was significantly correlated with breast muscle fat content, and expressed in other tissues [2]. Thus, it was speculated that the L-FABP gene may be the main gene that affects pig intramuscular fat content or a marker gene closely linked to the main gene [3].”

*[1] Xiong Xun, Li Yong, Ruan Yong, et al. Molecular characteristics and tissue expression analysis of FABP1 and FABP2 genes in pigs [J]. Southern Agricultural Journal, 2022, 53(4):11.*

*[2] He Jun. Correlation between L-FABP, A-FABP gene polymorphisms and intramuscular fat content in ducks and their effects on the expression of genes related to fat metabolism [D]. Zhejiang University, 2013.*

*[3] Cui Lu, Chen Xing, Zhou Ying, et al. Research progress of FABPs family genes [J]. Animal Husbandry and Veterinary Medicine, 2016(7):4.*

Introduction
-The first citation starts in 3. Please review the references and citations.

**Responses from the authors**: *Thanks for this comment. The reference list has been checked and fixed.*

-Why FABP1 was chosen to be evaluated instead of other known muscle/adipocyte FABPs isoforms? Previous studies have reported FABP1 as a predominant isoform in liver. If there is any evidence reporting FABP1 in muscle/adipocytes I suggest adding such information in introduction and/or discussion section.

**Responses from the authors**: *Thanks for the comment. To our knowledge, FABP1 and FAPB2 genes are two FABPs genes discovered earlier, which play an important role in the regulation of fat metabolism [1], and FABP1 gene is expressed in various tissues of pigs. He Jun et al [2] showed that duck L-FABP (FAPB1) gene polymorphism was significantly correlated with breast muscle fat content, expressed in other tissues, and played an important regulatory role in fat metabolism; thus, it could affect a variety of expression of fat metabolism-related genes. Cui Lu et al. [3] speculated that the L-FABP gene may be the main gene that affects pig intramuscular fat content or a marker gene closely linked to the main gene. Therefore, in this study, we selected FABP1.*

*[1] Xiong Xun, Li Yong, Ruan Yong, et al. Molecular characteristics and tissue expression analysis of pig FABP1 and FABP2 genes. Southern Agricultural Journal, 2022, 53(4):11.*

*[2] He Jun. Correlation between L-FABP, A-FABP gene polymorphisms and intramuscular fat content in ducks and their effects on the expression of genes related to fat metabolism. Zhejiang University, 2013.*

*[3] Cui Lu, Chen Xing, Zhou Ying, et al. Research progress of FABPs family genes. Animal Husbandry and Veterinary Medicine, 2016(7):4.*

L42-43: change “that is synthesized in large amounts” to “extensively synthesized”

**Responses from the authors**: *Thanks for this suggestion.* *Per your comment, it has been changed according.*

L44- 47: change “reservoir of” to “storage of”

**Responses from the authors**: *Per suggestion, it has been changed according.*

L52-55: This paragraph needs to be improved and the aim of the study need to be consisting to what was evaluated. The current study does not provide sufficient molecular and genetic basis for such affirmation.

**Responses from the authors**: *Thanks for your comment. In this study, the expression levels of FABP1 and FAS genes in the longissimus dorsi and subcutaneous adipose tissue of Zongdihua pigs were detected by qPCR technology, and the eukaryotic expression vectors of pEGFP-C1-FAS and pEGFP-C1-FABP1 were constructed to detect the difference between FABP1 and FAS. The role of collagen and its pyridine cross-linking, to explore the mechanism of fat deposition on collagen and cross-linking, and to provide a molecular theoretical basis for improving the resources of Zongdihua pig breeds.*

*Per your suggestion and those of other reviewers, we have added more background information in the Introduction section in the revised manuscript.*

Material and methods
L58: Typo “Statement”

**Responses from the authors**: *Corrected. Thanks.*

L64: Please specify how many pigs were used in each age.

**Responses from the authors**: *There were 6 for each age group. This information has been edited in the revised manuscript.*

L65: Correct the name of pigs’ breed

**Responses from the authors**: *Thanks for the comment. We have added the breed (Zongdihua pig) in the revised manuscript.*

L68: “…, frozen in liquid nitrogen and stored at -80 °C to further analyses.”

**Responses from the authors**: *Thanks for the comment, and we have changed it in the text.*

L69, L81: Please unify the information from those sections to its respective analysis

**Responses from the authors**: *We have revised it accordingly. Thanks.*

L88: Which shaded areas?

**Responses from the authors**: *They refer to shaded nucleotides of primer sequences of FABP1-CDS and FAS-CDS in Table 1. The revised manuscript reflects the changes accordingly.*

L102: Please cite the reference of this method

**Responses from the authors**: *Thanks, and we added a reference for this.*

*[4] Yang Yang, Xu Houqiang, Chen Wei, et al. Construction of Congjiang Xiang pig FGF10 gene interference vector and analysis of related gene expression. Journal of Agricultural Biotechnology, 2018, 26(12):9.*

L105: Subcutaneous fat cDNA from which pigs? 3d and 9m pigs?

**Responses from the authors**: *They were from the 3-day old pigs. It has been added to the text.*

L109: Please specify here the name of the commercial kit

**Responses from the authors**: *Thanks for this comment. We have added the information in the text.*

L118: How many 3d old pigs were used? The previous analysis (genes expression) was done with 3d and 9m old pigs? Please provide an explanation about the use of 3 d old pigs for adipocytes culture instead of 9m old pigs

**Responses from the authors**: *Thanks for the comments. There were six 3-day-old pigs used in the study. In the gene expression study, we used six of 9-month-old pigs. Since cells from younger pigs (such as 3d pigs) have better proliferation and differentiation ability, we used 9-month-old pigs for adipocytes culture, as they are more matured and have less cell proliferation and differentiation ability, yielding fewer variable cultures.*

L145: Please specify here the name of the commercial kit Please avoid the use of “(see below)” and “(see above)” throughout the text.

**Responses from the authors**: *Thanks for the comments. Our points are well taken. We have fixed them in the revised manuscript.*

L148: the section 1.5 can be placed together with this section

**Responses from the authors**: *Thanks for this comment. Now, section 1.9 has been merged to section 1.5.*

L153: Please add the statistical model in this section.

**Responses from the authors**: *Thanks for the comment. The 2-△△Ct statistic method was used to analyze the experimental data, and SPSS 20.0 software was used to analyze the significance level. This has been clarified in the text.*

L154: Please cite the method used to normalize mRNA.

**Responses from the authors**: *Thanks for the comment. For the method used for normalizing mRNA, we have added the following reference in the text.*

*Yang Yang, Xu Houqiang, Chen Wei, et al. Construction of Congjiang Xiang pig FGF10 gene interference vector and analysis of related gene expression. Journal of Agricultural Biotechnology, 2018, 26(12):9.*

L157: Why DNA sequencing was performed? Please specify and add the methods and steps of this analysis.

**Responses from the authors**: *Thanks for the comments. DNA sequencing was mainly to verify* *whether the eukaryotic vector was successfully constructed. Per your suggestion, we have added the information to the text.*

Results
L159: Please remove “analysis” from the section’s title

**Responses from the authors**: *Thanks for the comments. Done, it is removed.*

L169-172: Please indicate what was the purpose of these analyses

**Responses from the authors**: *The purpose of comparing the cloned fragment with the original sequence was to verify that the cloned fragment is the desired fragment or the correct fragment. Additionally, the bioinformatics analysis (original lines 170-172) was mainly to predict the physicochemical properties of FAS proteins and to understand the locality of Guizhou porcine FAS protein structure*

Figure 4: Please verify figure’s letters

**Responses from the authors**: *Thanks for the reminder. They are checked in the revised manuscript.*

L172-174: Please explain how this analysis was performed?

**Responses from the authors**: *Analysis of the secondary structure and physicochemical properties of FAS and FABP1 proteins were* *performed using the Protean program in DNAstar software. The text in the revised manuscript is changed to reflect this.*

L179-L181: Please correct the figure citation.

**Responses from the authors**: *Thanks for this comment. They are checked.*

Figure 6: intramuscular or subcutaneous preadipocytes?

**Responses from the authors**: *It referred to subcutaneous preadipocytes*.

L195: “…were constructed and fused to …”

**Responses from the authors**: *Thanks for the comment. It is fixed in the text.*

L199 and figure 7: what would be the blank control? please specify.

**Responses from the authors**: *Good comment.* *Blank control refers to the group of cells not transfected with pEGFP-C1-FAS and pEGFP-C1-FABP1 eukaryotic vectors. This has been reflected in the revised manuscript.*

Fig 8: Please correct figure legend “...transfected with pEGFP-C1-FAS and pEGFP-C1-FAS as indicated...”

**Responses from the authors**: *Thanks for the comment. It is modified in the figure legend.*

L204 and Figure 8: Please specify what would be the control group.

**Responses from the authors**: *Thanks for the comment. In the figure, the data from control group are shown in the bar on the left of each gene product, as they are marked in red below.*

L208-209: Did you mean double-antibody sandwich ELISA method?

**Responses from the authors**: *Thanks for the comment. Yes, it is ELISA method.*

Discussion
L217-218: Is there any other reference to cite this statement? I could not find reference number. Also, the “adipocyte-type fatty acid binding protein” in the title referes to A-FABP or FABP4 and not FABP1.

**Responses from the authors**: *Thanks for the good comment. We have added the following reference.*

*Ai Jinxin, Long Anju, Luo Weixing, et al. Study on the expression levels of FABP1 and FABP3 genes in different tissues of Qianbei hemp sheep. China Animal Husbandry, 2020, 56(11):68-72+78*

L219-220: Reference number 14 did not evaluate FABP1 expression

**Responses from the authors**: *Thanks for pointing this out. We have replaced it with the following two references:*

*1）Chen Guilian. Genetic polymorphism of pig L-FABP gene and its correlation with intramuscular fat content. Hebei Agricultural University, 2008.*

*2）Gao Yan, Zhang Yonghong, Xu Yanli , et al. Exon 2 polymorphism of L-FABP gene in Songliao black pigs and its correlation with meat quality traits. Chinese Journal of Veterinary Medicine, 2010, 30(8):4.*

L220-221: Is there any other reference to cite this statement? I could not find reference number 15。 Please carefully revise the discussion section and if possible, change the references that are not written in English, so the reader can easily find in the future

**Responses from the authors***: Thanks for the comment. We have made sure that original reference 15 is listed in the revised manuscript, which is:*

*Ai Jinxin, Long Anju, Luo Weixing, et al. Study on gene expression levels of FABP1 and FABP3 in different tissues of Qianbei hemp sheep [J]. Chinese Journal of Animal Husbandry, 2020, 56(11):68- 72+78. It is 18 in the revised manuscript.*

L226-228: This is a strong affirmative that need to be rewritten. Since the current study did not evaluate the protein levels of FABP1 and FAS or used methods to inactive the function of these proteins, there is no assurance that FABP1 and FAS can be considered the only candidate genes to infer in fat accumulation.

**Responses from the authors**: *Thanks for the comment. We agree with your point and made a modification to the sentence in the revised manuscript.*

L228-230: The results of this analysis need to be shown and better explained, as well as the methodology.

**Responses from the authors**: *Thanks for the comment. Per your suggestion, we added the following in the Discussion section, which reads:*

*In this study, the expression levels of FABP1 and FAS genes in the muscle and adipose tissue of Zongdihua pigs were detected by qRT-PCR method. muscle (P<0.01), which is the same as the previous research results, and the expression specificity tends to be consistent [18-19]. It indicated that FABP1 and FAS could be used as candidate genes for fat deposition. The higher the expression level, the stronger the fat deposition ability. Subsequently, DNAstar software was used to analyze the FABP1 and FAS proteins of Zongdihua pigs. Therefore, we used subcutaneous adipose tissue to culture primary adipocytes for more targeted and comprehensive experiments.*

L232: Please cite the previous studies.

**Responses from the authors**: *Thanks for pointing this out. References are added (25).*

L232: “....that COL3A1 and LOX…”

**Responses from the authors**: *Thanks for the comment. Together with consideration of another review, we have deleted LOX.*

L233: “…than longissimus dorsi muscle ...”

**Responses from the authors**: *Similar to your comment immediately above, we have fixed this part in the revised manuscript.*

L232-233: That is not what was reported in Figure 1, where the expression of all the genes were higher in subcutaneous fat compared to muscle.

**Responses from the authors**: *Thanks for the comment. It is a very good catch. The sentence now is revised as you suggested.*

L234: Please add a reference to the statement.

**Responses from the authors**: *Thanks for the comment. We have added the following reference that its finding showed that the expression level of COL3A1 gene in subcutaneous fat was significantly higher than that in longissimus dorsi muscle tissue.*

*Yang Rong, Zhou Di, Yan Zhihong, et al. Research on the characteristics of local pig collagen in Guizhou and the tissue expression of related genes [J]. Chinese Journal of Animal Husbandry, 2021, 57(8):5:*

L236: What did you mean with “may damage”? Please provide better explanation

**Responses from the authors**: *Thanks for the comment. We meant to say that fat deposits may disrupt the structure of connective tissue. It has been modified in the revised manuscript.*

L243: Typo “adipocytes”

**Responses from the authors**: *Thanks for pointing out the typo. It has been corrected.*

L244: Which gene products?

**Responses from the authors**: *Thanks for the comment. It refers to FAS gene products, as FAS gene expression can affect fat deposition and breakdown. We have clarified in the text.*

What did you mean with “decomposition of fat”. Please be more objective.

**Responses from the authors**: *Thanks for the comment. It is because* *that FAS gene is believed a key gene in the process of fat deposition, and its expression can affect fat deposition. Over-expression of FAS gene can promote cell proliferation, inhibit cell apoptosis, and also affect the synthesis of triglycerides in adipocytes, thereby affecting fat deposition. We have incorporated some of these in the revised manuscript.*

L253: Add citation

**Responses from the authors**: *Thanks for the comment. References are added in L301-303 (i.e., #32-34 in the revised manuscript).*

L262: Is there any refence reporting the meat quality parameter of Zongdihua pigs comparing with other breeds? If so, it could be included to support this hypothesis.

**Responses from the authors**: *Thanks for the excellent comment.* Yes, a previous s*study has shown that the skin thickness of Zongdihua pigs is as high as 0.55cm, which is higher than that of Guanling pigs and Jiangkou radish pigs [x], and the tenderness shear force value is 2.925 kg. Thus, we have added the reference in the revised manuscript.*

*Feng Wenhao. Study on genetic diversity and genetic relationship of three local pig breeds in Guizhou. Guizhou University, 2008.*

Conclusions
As previously mentioned, this affirmation should be excluded or reformulated. Moreover, a perspective of future studies could be included.

**Responses from the authors**: *Thanks for the comment. We have added future plan in the Conclusion section.*

Figures

I suggest reporting Figures 2, 5, 6 , and 7 as supplementary.

**Responses from the authors**: *Thanks for your comments, and we have the revision in the revised manuscript according to your suggestion.*

**Reviewer #2**:
The title reflects the work's main result which means that instead of “Zongdihua pig tissues” you must use Zongdihua pig isolated adipocytes once the overexpression was done in isolated cells, not in tissue.

**Responses from the authors**: *Thanks for this comment. We have replaced the word “tissues” with “isolated cells”.*

ABSTRACT
Line 19. Please, Replace Zongdihua pig by Zongdihua pig isolated adipocyte
Line 19. Replace identify processes by identify biochemical processes
Line 20. Replace, using molecular genetics by molecular tools

**Responses from the authors**: *Thanks for these suggestions. They have been corrected in the revised manuscript.*

INTRODUCTION
Line 40. In the first paragraph, please, develop in a deeper way the signaling pathway crosslink between the genes FABP1, FAS, and COL3A1.

**Responses from the authors**: *Thanks for the excellent point. Per your suggestion, we have added the following into the Introduction section.*

*The extracellular matrix (ECM) can protect mature adipocytes from being destroyed by external compression and stimulation, and the main component of ECM is collagen [1-2]. Studies have shown that in the process of fat deposition, a large amount of collagen is synthesized [3]. TGF-β positively regulates genes encoding collagen [4], and these collagen-encoding genes are also highly expressed in tissues with higher fat content [5]. In the study of the effect of splicing enzyme (ADAMTS2) in the process of collagen formation on intramuscular fat, it was found that the content of intramuscular fat was significantly positively correlated with the expression of ADAMTS2 gene. It can be seen that there is an intrinsic link between collagen and fat, but the specific regulatory mechanism remains unclear.*

Line 53. Please, in the sentence “In the present study… you should increment the paragraph with a clear objective of the study. Which question will be answered?

**Responses from the authors**: *Thanks for the excellent comment.* *Per your suggestion, we have added some in the Instruction section to clearly lay out the objective.*

MATERIAL AND METHODS
Line 87. Please, add the Accession number (e.g NM_) of the sequences you have used to design oligonucleotides.

**Responses from the authors:** *Thanks for the comment. L83-86 provided FAS, FABP1, GAPDH accession numbers. Additionally,* *the accession number for LOX gene is NM_001206403 and COL3A1 is NM_001243297, which is also added in the text.*

Line 138. If you have used 4 ug of plasmid to transfect, please add this information just before the sentence “recombinant plasmids pEGFP-C1-FABP1 and pEGFP-C1-FAS”. Otherwise, what is the 4ug of DNA? Add empty plasmids controls and blanks to sentence. Clarify this sentence until line 140.

**Responses from the authors**: *Thanks for the comment. Per your suggestion, the text has been revised in the manuscript.*

Line 153. In Data processing and analysis, the 2-△△Ct method is a gene expression method and I do not identify the statistical methods used not only in gene expression analysis, but also in the other analysis. Please, add the proper information and experimental design in this subtopic

**Responses from the authors**: *Thanks for the comment. 2-△△Ct is the statistical method, and it was used to statistically analyze the experimental data. We have clarified in the text.*

RESULTS AND ANALYSIS
Line 163. Please, delete the term “extremely”

**Responses from the authors**: *Thanks for the comment. It has been deleted.*

Line184. Delete the term “and differentiate” or changed it to “morphology differentiate” following the idea of the next sentence.

**Responses from the authors**: *Thanks for the comment. The phrase “and differentiate” have been deleted.*

Line 191. Don’t you have the quantification the lipids content staining with Oil Red O as you mentioned in MM? Put the data in figure 6 and refer to it here.

**Responses from the authors**: *Thanks for the comment. Oil red O staining was used to identify adipocytes. The prepared oil red O stock solution and distilled water were mixed at a ratio of 3:2 to form an oil red O working solution, and the working solution was added for staining for 1 h. It was not used as a quantitative measurement.*

Line 195. Please, the sentence “Cloned versions of FAS and FABP1 were constructed that were fused to the eGFP protein at their carboxyl termini and over-expressed via the strong CMV promoter” is important to MM section. Relocate it. Otherwise, just here the reader will understand what was done.

**Responses from the authors**: *Thanks for the comment. This information has been moved to MM section under 1.6.*

FIGURES
Figures 1 and 8. Please, in the Y axis add “/GAPDH” just after relative expression.

**Responses from the authors**: *Thanks for the comment. Your suggestion is accepted and fixed in the figures.*

Figures 5 and 6. Please, add magnification bar in the bottom corner of the image Images seems in different magnification.

**Responses from the authors**: *Thanks for your comment. Although it appears different in magnifications, they are indeed the same. We have inserted a magnification bar (100 x) in the bottom of the image in both figures.*

Figure 7. Here we have some problems. This figure per se is not a result, but only a control of the efficiency of you transfection. I strongly recommend you to put together this figure to figure 8. In addition, I think we have a problem with the image once only pEGFP-C1-FAS transfected cell have been shown (doubled data). The magnification of the images seems different, please, correct that.

**Responses from the authors**: *The magnifications for Figures 7 and 8 are same. Reviewer one also has comments on those figures. Per your suggestion as well as the other reviewer, we have adjustments in figures to accommodate both of your comments. Thanks again for the excellent comments.*

**Reviewer #3:**

The manuscript entitled “Fatty acid binding protein 1 and fatty acid synthetase overexpression have differential effects on collagen III and cross-linking in Zongdihua pig tissues” aims to determine whether FABP1 and FAS regulate expression of collagen and its crosslinking via lysyl oxidase in Zongdihua pigs. The manuscript points out that enhancing the expression of FAS and FABP1 increases collagen accumulation and this preliminarily suggests that FAS and FABP1 can serve as fat-related candidate genes providing a theoretical basis for the study of fat deposition in Zongdihua pigs. The manuscript brings interesting results, however, to be accepted for publication, it needs major changes and corrections. Below are suggestions to make the manuscript suitable for publication and more informative to the reader.

**Responses from the authors**: *Thanks for the comments. We are committed to work with you to improve the manuscript.*

General suggestions
- Write all gene names in italics. Example: FABP1

**Responses from the authors**: *Thanks for the comment. We have checked and corrected them.*

- Please review the text for language.

**Responses from the authors**: *The revised manuscript has been proofread by a native English speaker.*

- Some words are used in different ways in the manuscript such as cross-linking/crosslinking, Over-expression/Overexpression…Correct this, please.

**Responses from the authors**: *Thanks for the comment. The word has been corrected. Also, the revised manuscript has been proofread by a native English speaker.*

- The introduction needs to be rewritten, addressing the issues related to the manuscript more deeply. In addition, give a clear hypothesis and objective in the introduction section.

**Responses from the authors**: *Thanks for your comment. We have added some regarding the pig breed and why it is a good candidate.*

- Please, change “longissimus dorsi” to “Longissimus dorsi” throughout the manuscript.

**Responses from the authors**: *Thanks, and we have changed them accordingly.*

- Discussion needs to be rewritten, addressing more deeply the observed results.

**Responses from the authors**: *Thanks for your comments. Per your suggestion, we have revised some parts in the Discussion section.*

Abstract
- Line 26 - Remove the with in the sentence "FAS encodes 333 amino acid hydrophobic protein containing with 26 phosphorylation sites and 0 transmembrane regions".

**Responses from the authors**: *Thanks for the comment. It is corrected in the revised manuscript.*

- Line 27 - Change the following sentence "The basal levels of FABP1 and FAS in pig tissues expression were 3 -3.5-fold higher in subcutaneous fat compared with muscle (P < 0.01)" to "The basal levels of FABP1 and FAS expression in pig tissues were 3 -3.5-fold higher in subcutaneous fat compared with muscle (P < 0.01)".

**Responses from the authors**: *Thanks for the comment. The sentence is corrected*.

- Line 33- I believe you forgot to add “FAS” in this sentence "Therefore, enhancing FABP1 expression increases collagen accumulation...". Am I correct? Because the overexpression of FAS and FABP1 resulted in a significantly increased expression of the COL3A1 gene.

**Responses from the authors**: *Thanks for the comments. Yes, you are correct, and the missing word “FAS” has been added.*

- Keywords must be arranged in alphabetical order. It is not recommended to add words that are already in the title of the manuscript such as Zongdihua pig.

**Responses from the authors**: *Thanks. They are corrected per your suggestion.*

Introduction
- Add the title “Introduction” before the text in the line 40.

**Responses from the authors**: *Yes, it is added.*

- You could improve your introduction by talking more about the breed (Zongdihua pig), mainly about the meat traits.

**Responses from the authors**: *Thanks for your comment. We have added the following in the Introduction section, which reads:*

*Zongdi Huazhu is mainly produced in Zongdi Town, Guizhou Province. It is one of the local pig breeds in Guizhou with excellent meat quality. It has good motherhood, strong adaptability and disease resistance. The meat is fragrant and glutinous, with the meat quality characteristics of high-grade meat products. Zongdihua pigs are fatty pigs with a lean meat rate of only 38%. Studies have shown that Zongdi Hua pork has better quality properties than large white pigs [8].*

*[8] Zhang Yun, Chen Wei, Li Ping, et al. Carcass and meat quality traits of land-colored pigs with different rearing methods. Guizhou Agricultural Science, 2014, 42(9):3.*

It could also present some preliminary studies that address the evaluated genes deeply. All these issues need to be better integrated. In addition, the objective of the work should be better presented in this introduction.

**Responses from the authors**: *Thanks for the comments. No, sorry that we did not have additional study data to add. Due to strict Covid-19 control policy in China, adding new lab studies at this time is extremely difficult. However, we will keep this in mind and will incorporate the suggestion to our future studies.*

Materials and methods
- Line 58 - Correct to Ethics Statements.

**Responses from the authors**: *Thanks. It is fixed.*

- Why is the topic "Ethics Statements" highlighted in the text?

**Responses from the authors**: *We have removed the highlight.*

- Please, I ask you to correct the text in relation to the language used. Some sentences are poorly worded.

**Responses from the authors**: Thanks for the comments. We have our revised manuscript checked by a native English speaker.

- You don't write about the design and accession number of the following genes: LOX and COL3A1 in the text.

**Responses from the authors**: *Thanks for the comments. Per suggestion, we have added the information, i.e., LOX gene: NM_001206403; COL3A1: NM_001243297*

- Did you work with the fold change (comparative CT method)? Why are the graphs with the Y-axis legend as a relative expression? I suggest reading the article "Analyzing real-time PCR data by the comparative CT method - Thomas D Schmittgen1 & Kenneth J Livak 2008" to correct this question

**Responses from the authors**: *Thanks for the comment. The method used in this study was to present relative gene expression; thus, it is a comparative C(T) method. The Y-axis of the graph represents the calculated relative expression of the gene.*

- Subcutaneous fat and Longissimus dorsi samples were collected from how many animals?

**Responses from the authors**: *Thanks for your comment. Subcutaneous fat and muscle tissues were collected from 6 9-month-old pigs*

What is the N of this gene expression analysis? You need to better describe the experimental design of the study.

**Responses from the authors**: *Thanks for the comment. Not quite clear about the comment. Is this regarding mRNA expression analysis? Based on other comments, we have added some in the section.*

Results
- Why in the legend of figure 1 you used fat deposition if there is only information about the fold change between subcutaneous fat and Longissimus dorsi?

**Responses from the authors**: *Thanks for the comment.* *Fat deposition in Figure 1 does not refer to tissue, the legend means more about the expression of fat deposition-related genes (FABP1 and FAS) and collagen-related genes (LOX or COL3A1) in porcine subcutaneous fat and muscle tissue.*

- Line 163:164 – “The expression levels for all 4 genes were extremely significantly (P < 0.01) elevated in subcutaneous fat tissues compared with the muscle (control) tissues (Figure 1).” Why did you compare subcutaneous fat to muscle?

**Responses from the authors**: *Thanks for the comment.* *Zongdihua pigs are fat-rich pigs, they have high fat content with thick back fat and thick skin. Also, because their meat is chewy and full of glutinous aroma, their meat quality is generally considered good. Therefore, in order to explore the characteristics of Zongdihua pigs, the subcutaneous fat and muscle are selected tissue targets for comparison.*

Your hypothesis should be very clear in the manuscript in relation to the comparisons made. Besides that, could you explain a little more about what you considered as a control in this study?

**Responses from the authors**: *Thanks for your comment. In this study, FABP1 and FAS genes were highly expressed in adipocytes, which helps verifying whether high expression of FABP1 and FAS genes would affect the expression of collagen-related genes, and it further helps exploring the mechanism of fat deposition on collagen and cross-linking.*

- Is the Y-axis legend correct in Figure 9?

**Responses from the authors**: Thanks for the comment. *Yes, we have double checked and it is correct.*

Discussion
- Line 225:226 – Change the sentence “In this study, we found that FABP1 and FAS gene expression were significantly higher than for the control muscle tissue” to “In this study, we found that FABP1 and FAS gene expression were significantly higher in subcutaneous fat than for the control muscle tissue”

**Responses from the authors**: *Thanks for the comment, and we have replaced the sentence per your suggestion.*

- Line 232 – Alter LOC gene to LOX.

**Responses from the authors**: *Thanks, and this is fixed. We also, searched the entire manuscript to make sure that there is no LOC.*

- Your discussion of the results is superficial, you need to go deeper into the effect of the FAS and FABP1 genes on the expression of COL3A1 and LOX and the production of collagen. In addition to the effects of these genes on the deposition of fat and on meat traits.

**Responses from the authors**:

- Line 231:233- “Our results agreed with previous studies indicating that COL3A1 and LOC gene expression in subcutaneous fat are significantly higher and lower than in longissimus dorsi muscle tissues, respectively.” - Figure 1 shows greater expression of both the COL3A1 and LOX genes in subcutaneous fat in relation to Longissimus dorsi.

**Responses from the authors**: *Thanks for the comments. It was our mistake. Now, the sentence in the text only addresses COL3A1, so it matches with the result showing in Figure 1.*

- You have not discussed deeply the effects of the expression of these genes on meat quality in the breed studied.

**Responses from the authors**: *Thanks for your comment! Per your suggestion, we have added the following in the Discussion section.*

*After overexpression of FAS and FABP1 genes, the expression of COL3A1 increased and the expression of LOX decreased, indicating that with the deposition of fat, collagen synthesis increased, while the expression of LOX was inhibited, resulting in a decrease in mature cross-linking content. Therefore, the high expression of these genes makes Zongdihua pigs high in fat and collagen, but low in cross-linking, resulting in thick and waxy skin and better meat tenderness.*
