# Supplementary material for: Fatty acid binding protein 1 and fatty acid synthetase over-expression have differential effects on collagen III synthesis and cross-linking in Zongdihua pig primary adipocytes
Source: PLoS One. 2023 May 4;18(5):e0270614. doi: 10.1371/journal.pone.0270614 (PMC10159151; doi:10.1371/journal.pone.0270614)
Supplement: Attachment — Submitted filename: Responses to editor.docx [file pone.0270614.s009.docx]

**Responses to Reviewers’ comments**

Dear Editors and Reviewers:

Thank you for your letter and for the reviewers’ comments concerning our manuscript entitled“**Fatty acid binding protein 1 and fatty acid synthetase over-expression have differential effects on collagen III synthesis and cross-linking in Zongdihua pig primary adipocytes”(ID:PONE-D-22-15349R1)**. Those comments are all valuable and very helpful for revising and improving our paper, as well as the important guiding significance to our researches. We have studied comments carefully and have made correction which we hope meet with approval. Revised portion are marked in yellow on the revised version. The main corrections in the paper and the responds to the reviewer’s comments are as flowing:

Responds to the reviewer’s comments:

Reviewer #1:

Title: Fatty acid binding protein 1 and fatty acid synthetase over-expression have differential effects on collagen III synthesis and cross-linking in Zongdihua pig primary adipocytes.I have noticed some changes in the current manuscript version, however, some suggestions and questions were answered but not addressed in the manuscript. The general introduction and discussion need to be restructured to provide a better link between the paragraphs and a better understanding of the reader. Please consider the suggestions bellow:

**Abstract**

1. L30-31: “We aimed…”

**Responses from the authors:** *Thanks for your excellent suggestions.Modified according to your suggestions.*

2.L32: breeding improvement in terms of what?

**Responses from the authors:** *Through the research on the meat quality characteristics of the Landflower pig, the dominant traits of the breed can be excavated, and the traits such as slow growth, small size and low reproduction rate can be improved while retaining these excellent traits in the future breeding process.*

3.L37: Please remove “FAS encodes …….regions.”

**Responses from the authors:** *Thanks for this comment.We have deleted as required.*

**Introduction**

4.L52- 70: These two paragraphs need extensive revision mainly in term of sentence structure. For example, L52-55 can be transformed into 3 sentences “The extracellular matrix (ECM) play roles in adipocyte proliferation, differentiation, and migration. Moreover, ECM anchors these cells to prevent mechanical movement and protect mature adipocytes from external compression and stimulation. It is well established that collagen is the main component of ECM ….”

**Responses from the authors:** *Thanks for your excellent suggestions.Your points are well taken. Per your suggestion, in the revised manuscript,We revised the sentence structure.L52-55 is modified as follows:The extracellular matrix (ECM) play roles in adipocyte proliferation, differentiation, and migration. Moreover, ECM anchors these cells to prevent mechanical movement and protect mature adipocytes from external compression and stimulation. It is well established that collagen is the main component of ECM*

5.L81-85 This paragraph is disconnected from the rest of the text, please reorganize the introduction section. I would suggest to focus and organize this section by following the topics: Breed and meat characteristics, ECM and its components, FABP1 and it regulatory role, and a clear description of the objectives (The present study aimed to investigate x, y and z …)

**Responses from the authors:** *Thanks for your excellent summary comments. Your points are well taken. Per your suggestion,We have modified and supplemented this part.*

L81-85 *The revised contents are as follows:Zongdihua pig is mainly produced in Zongdi Township, Ziyun County, Guizhou Province. It has strong disease resistance, tolerance to roughage, and a strong ability to digest and absorb crude fiber. Its meat features excellent quality, bright red flesh color, rich intramuscular fat, high water tying rate, little cooking loss, thick carcass skin, soft and waxy texture, easy to cook, and tender and fragrant muscles, which is highly appreciated by consumers. It is a valuable local genetic resource with a high value of development and utilization [11, 16]. As mentioned above, collagen is the main component of ECM, which has an important influence on the tenderness, viscoelasticity and chewiness of the meat, and it will be synthesized in large quantities when fat is deposited. However, Zongdihua pig is a fatty breed, and its lean meat rate is only 38%. In addition, the study shows that the polymorphism of the FABP1 gene is significantly related to the fat content of chest muscle, which is expressed in various tissues of pigs. It is speculated that the FABP1 gene may be the main gene affecting the intramuscular fat content of pigs, or a marker gene closely related to the main gene. Therefore,the present study aimed to investigate the effects of FABP1 and FAS on the cross-linking of collagen and pyridine, and provide a molecular theoretical basis for the development and utilization of local pig genetic resources.*

**Material and Methods**

6.L95: “Healthy Zongdihua pigs including …”

**Responses from the authors:** *Thanks for this comment.Healthy Zongdihua pigs including conform to the characteristics of this breed, have good growth performance and have no diseases.*

7.Section 1.3 (Reagents) and 1.4 (primer design and synthesis): the information in these sections should be placed with its respective analyses and these topics removed, as I have already mentioned in the first revision.The accession numbers described in section 1.4 could be better visualized if placed in a column of Table 1.

**Responses from the authors:** *Thanks for this suggestion. Per your comment, it has been changed according.The information in 1.3 (Agents) and 1.4 (print design and synthesis) has been put together with the corresponding analysis, and these topics have been deleted. At the same time, in order to be more intuitive, the accession number described in Section 1.4 has been placed in Table 1.(Red font)*

**Table 1. PCR primers used in this study**

| Gene | Numbers | Sequence (5’→3’） | Annealing temperature/℃ | Product length/bp | [Purpose](D:/QQ%20%20%E6%96%87%E4%BB%B6%E4%B8%8B%E8%BD%BD%20%E5%A4%84/Youdao/Dict/8.9.3.0/resultui/html/index.html#/javascript:;) |
| --- | --- | --- | --- | --- | --- |
| *FABP*1 | NM_001004046.2 | F:TTGGGAGAGGAGTGTGAGATG  R:CACAGACTTGATGCCTTTGAA | 58 | 111 | qPCR |
| *FAS* | NM_213839.1 | F:CCTGTATCGCTGGACCACT  R:GGGCACTCAGACTCCCTTT | 57.9 | 103 | qPCR |
| *GAPDH* | XM_021091114.1 | F:TTGTGATGGGCGTGAACC  R:GTCTTCTGGGTGGCAGTGAT | 60.0 | 169 | qPCR |
| *LOX* | NM_001206403 | F:TACCAAGCCGACCAAGATA '  R:GTGTGCAGTACAGGCAAAT | 57.2 | 147 | qPCR |
| *COL*3*A*1 | NM_001243297 | F:TTTGCTCTACTTCATCCCACT  R:CTTCCAGACATCTCTATCCGC | 56.5 | 105 | qPCR |
| *FABP*1-CDS | NM_001004046.2 | F: CCGATGAACTTCTCCGGCAAATACC  R:CACGGTACCCTCTTGCTGATTCTCTTGAAGACAA | 58.5 | 384 | Cloning |
| *FAS*-CDS | NM_213839.1 | F:CCGATGTCCGGGATCTGGGTTCT  R:CACGGTACCCTAGGTCAAGCTTTCATTCTCATTC | 60.0 | 999 | Cloning |

8.L125: The total RNA was extracted from fat and longissimus dorsi muscle from pigs in which age? 3d , 9 mo or both? Since you have collected tissue from both ages and in some analysis you used only one age, make sure you are being specific throughout the manuscript.

**Responses from the authors:** *Thanks for the comment.Extraction of total RNA from fat and longissimus dorsi muscle of 9 month old pigs. This information has been edited in the revised manuscript.*

9.L155: by DNA sequencing? How was done the bioinformatic analysis of DNA sequencing data? Please add a description or reference of the employed methodology.

**Responses from the authors:** *Thanks for the comment.A comparison of the DNA sequencing results in NCBI showed that the results were consistent with the original sequence. The physicochemical properties, secondary structures, transmembrane regions, and phosphorylation sites of FABP1 and FAS proteins are analyzed by the bioinformatics analysis software.The method adopted in the revised manuscript has been supplemented.*

10.L187: It is not clear what was considered in the statistical model, fixed and random effects. All the information needs to be clear to allow reproducibility.

**Responses from the authors:** *Thanks for this comment.Fixed effect model is considered to be used as the statistical model, which aims to compare the expression of related genes in different tissues in this experiment. These comparison objects are fixed, not randomly selected. We use a one-way analysis of variance (F-test) to choose the form of the data model.*

**Results**

11.L192: “2 Results”

**Responses from the authors:** *Thanks for this suggestion. Per your comment, it has been changed according.*

12.L193: “2.1 Differential expression analysis”

**Responses from the authors:** *Thanks for this suggestion. Per your comment, it has been changed according.*

13.L164: please specify the age of the pigs in text and Figures.

**Responses from the authors:** *Thanks for the comment. We indicated the age of pigs in the revised manuscript.*

14.L236: “… were examined for lysyl …”

**Responses from the authors:** *Per suggestion, it has been changed according.*

15.L239-240: “(Figure 5B)”

**Responses from the authors:** *Per your comment, it has been changed according.*

**Discussion**

16.L260-261: This is a strong affirmation. Since you compared two different cell lines (from muscle vs adipose tissue), it is expected, and already reported, greater influence of this genes on adipocytes compared to muscle. To ensure that these genes are associated with greater fat deposition ability you should have evaluated pigs with different levels of subcutaneous fat, different breeds, etc. Please rewrite the indicative of this data or provide references of the influence of these genes on greater subcutaneous fat deposition.

**Responses from the authors:** *Thanks for your excellent comment. Per your suggestion, we added the following in the Discussion section, which reads:*

*At the same time，alterations in the primary sequences that would affect enzymatic functions were not detected.The expression level of FABP1 and FAS in muscle and adipose tissue is different among breeds and within breeds. The expression level of subcutaneous adipose tissue in Kele pig and DLY crossbred pig is significantly higher than that in longissimus dorsi muscle, and the expression level of subcutaneous adipose FABP1 in different breeds is also different. The expression level of subcutaneous adipose FABP1 in the Kele pig is higher than that in the DLY crossbred pig, which may be due to the fact that the Kele pig is a local pig of a fatty breed with large fat deposition. In this study, the expression of FABP1 and FAS genes in the subcutaneous adipose tissue of Zongdihua pig is significantly higher than that of the longissimus dorsi muscle, which is similar to other pig breeds.*

17.L274-277: repeated sentences.Similar to introduction, the paragraphs seem disconnected. Please improve the link between paragraphs for better understanding and clarity.

**Responses from the authors:** *Good comment.Per your suggestion,we have changed it in the text:*

*The studies show that the expression of the COL3A1 gene in pig subcutaneous fat is significantly higher than that in longissimus dorsi muscle. Results were consistent with higher levels of collagen in fat compared with muscle tissues. The expression level of the LOX gene is lower than that in longissimus dorsi muscle, and the content of pyridine cross-linking (HP and LP) is the lowest in subcutaneous fat. We speculate that this might be related to fat deposition. We therefore cultured primary adipocytes from subcutaneous fat tissues in these pigs. We were able to successfully over-express FAS and FABP1 in Zongdihua pig preadipocytes and this resulted in increased COL3A1 and decreased LOX expression levels (P<0.01) as well as lysyl oxidase activities. These results indicate that fat deposition may disrupt the structure of connective tissues resulting in an increase in tenderness with age.*

18.Conclusion: ok

**Responses from the authors：***Thanks!We look forward to your "acceptance" suggestions and responses！*

19.Figure 3. Please verify the letters.

**Responses from the authors:** *Thanks for this comment. They are checked.*

20.Figure 4 “…. COL3A1..” ; transfected with (A) pEGFP-C1-FAS and (B) pEGFP-C1-FAS? Please correct figure’s legend.

**Responses from the authors:** *Thanks for this comment.It is modified in the figure legend:transfected with (A) pEGFP-C1-FABP1 and (B) pEGFP-C1-FAS.*

Reviewer #2:

Dear Authors,

Thank you to considering most of questions raised previously to make the manuscript suitable for publication and more informative to the reader.Most of concerns were properly addressed, but I still have some serius concerns about the Statistics.To make the data more informative, at the Data processing topic, 2^-ddCt is a method to access the fold relative gene expression. In order the access the differences in gene expression between the groups you must use a proper statistical model and test (parametric or non-parametric) and I still have not seen it in the text. Please, it is important to add not only the software, but also the test you have used. In addition, please add a supplementary table at the supplementary material with cT collected data from qPCR for each replicate, dissociation curves for each gene reaction and the statistical metrics.

**Responses from the authors:***Thanks for your excellent comments.The 2-△△Ct method also referred to as the comparative C T method.This method is used to calculate the relative quantification of target gene and reference gene, and compare with reference gene.Mathematical model for relative quantification in real- time PCR*

*The mathematical statistical model of relative quantification in real-time PCR of this test is calculated according to the method provided by Schmittgen T D and Michael*

*[1] Schmittgen T D . Analyzing real-time PCR data by the comparative CT method[J]. Nature Protocols, 2008.*

*[2]Michael, W, Pfaffl. A new mathematical model for relative quantification in real-time RT–PCR[J]. Nucl Acids Res, 2001.*

*In addition, add a table about your proposal, including each duplicate ct data collected from qPCR. Due to the outbreak of the COVID-19 in China, we cannot retrieve the computed CT data, but we have retained the dissociation curve and statistical indicators of each gene reaction in qPCR. We have added this part to the "Fig. S5 The sampling curve and measuring curve of (A) COL3A1, (B) LOX, (C) FAS, (D) FABP1, (E) GAPDH gene" in the supplementary materials*
