# Supplementary material for: Fatty acid binding protein 1 and fatty acid synthetase over-expression have differential effects on collagen III synthesis and cross-linking in Zongdihua pig primary adipocytes
Source: PLoS One. 2023 May 4;18(5):e0270614. doi: 10.1371/journal.pone.0270614 (PMC10159151; doi:10.1371/journal.pone.0270614)
Supplement: Attachment — Submitted filename: Responses to editor.docx [file pone.0270614.s010.docx]

**Responses to Reviewers’ comments**

Dear Editors and Reviewers:

Thank you for your letter and for the reviewers’ comments concerning our manuscript entitled“**Fatty acid binding protein 1 and fatty acid synthetase over-expression have differential effects on collagen III synthesis and cross-linking in Zongdihua pig primary adipocytes”(ID:PONE-D-22-15349R2)**. Those comments are all valuable and very helpful for revising and improving our paper, as well as the important guiding significance to our researches. We have studied comments carefully and have made correction which we hope meet with approval. Revised portion are marked in yellow on the revised version. The main corrections in the paper and the responds to the reviewer’s comments are as flowing:

Responds to the reviewer’s comments:

Reviewer #1:

The answers were addressed, and I could realize an improvement throughout the text. However, it still need some minor revision, mainly regarding the clarity in some sentences to make the paper more objective to the reader. Please consider the comments below.

**Abstract:** ok

**Responses from the authors：***Thanks!We look forward to your "acceptance" suggestions and responses！*

**Introduction**
L48-49: “…fibrillar collagens and the expression….”

**Responses from the authors:** *Thanks for your excellent suggestions.Modified according to your suggestions.*

L55: “…such as TGF-β, which is a positive…”

**Responses from the authors:** *Thanks for your excellent suggestions.Modified according to your suggestions.*

L56: “…collagen genes and it is highly…”

**Responses from the authors:** *Thanks for your excellent suggestions.Modified according to your suggestions.*
 L71-85: I suggest to remove this paragraph

**Responses from the authors:** *Thanks for this suggestion. Per your comment, it has been changed according*

**Material and Methods**
 L95-96: not necessary to report “( conform to the characteristics of this breed, have good growth performance and have no diseases)”. I suggest to remove

**Responses from the authors:** *Thanks for your excellent suggestions.We have removed it in the revised version.* L135: “…proteins were analyzed…”

**Responses from the authors:** *Per suggestion, it has been changed according.*

L144: Please remove “as per above” here and throughout the text

**Responses from the authors:** *Thanks for your excellent suggestions.We have removed it in the revised version.*
 L145: “The fat cell of 3-day-old pigs were cultured and the pellets were suspended…”

**Responses from the authors:** *Thanks for your excellent suggestions.Modified according to your suggestions.*
 L162: please correct “37oC”

**Responses from the authors:** *Per suggestion, it has been* corrected *according.*

**Results**
 L201: Please verify the Figure’s citation here and throughout the text

**Responses from the authors:** *Thanks for your excellent suggestions. Per your suggestion,We have modified and verified this part.*
 L224: Figure 4 shows the P < 0.01 for COL3A1 expression in both over-expressed clones. Please correct this information here or in the Figure

**Responses from the authors:** *Thanks for your excellent summary comments. Your points are well taken. Per your suggestion,We have modified and verified this part.After repeated verification, we have corrected Figure 4 and made correct modifications in the text.*

L226-227: Please correct this sentence for “We further examined the effects of transfected plasmids on LOX mRNA levels and lysyl oxidase activity.”
 **Responses from the authors:** *Per suggestion, it has been* corrected *according.*
 L229: “..double-antibody sandwich ELISA method…”
 **Responses from the authors:** *Thanks for this suggestion. Per your comment, it has been changed according.*

**Discussion**

L253-256: Please substitute the sentence to: “The expression level of subcutaneous adipose tissue in Kele pig and DLY crossbred pig is significantly higher than longissimus dorsi muscle” and add a citation.

**Responses from the authors:** *Thanks for your excellent summary comments. Per your suggestion,We have modified this part.*Dear Reviewer,*Because of the sentence It is the result of this experiment, so no citation is added.*

L256-258: Please substitute the sentence to: “Moreover, the expression level of subcutaneous adipose FABP1 in the Kele pig is higher than DLY crossbred pig, which may be due to the fact that the Kele pig is a local pig that present a high fat deposition” and add a citation.

**Responses from the authors:** *Thanks for your excellent suggestions.Modified according to your suggestions.*

L259: “…significantly higher compared to longissimus dorsi muscle…”

**Responses from the authors:** *Thanks for your excellent suggestions.Modified according to your suggestions.*

L260: “These data may indicate that FABP1….”

**Responses from the authors:** *Thanks for your excellent suggestions.Modified according to your suggestions.*

L263: which studies? Please cite

**Responses from the authors:** *Thanks for this comment.The* study *here refers to the author's previous research results, citation 29*

L264: “significantly higher compared to longissimus dorsi muscle. The results…”

**Responses from the authors:** *Thanks for your excellent suggestions.Modified according to your suggestions.*

L265: “….compared to muscle”

**Responses from the authors:** *Thanks for your excellent suggestions.Modified according to your suggestions.*

L265-267: The expression of LOX is lower in fat compared to muscle or the expression of LOX is lower in muscle compared to fat?? This sentence is confused. Please rephrase this sentence and cite the paper.

**Responses from the authors:** *Thanks for your excellent comment.We have modified this* sentence.

The expression of LOX is lower in fat compared to muscle, and the content of pyridine cross-linking (HP and LP) is the lowest in subcutaneous fat^[29]^.

L267-270: I suggest to substitute by “By culturing primary adipocyte from subcutaneous fat we were able to successfully over-express FAS and FABP1 in Zongdihua pig preadipocytes and this resulted in increased COL3A1 and decreased LOX expression levels (P<0.01) as well as lysyl oxidase activities”

**Responses from the authors:** *Thanks for your excellent suggestions.We have* substituted *this part*.

L277: “…gene products collectively affected…”

**Responses from the authors:** *Per suggestion, it has been changed according.*

L290: “…while inhibited LOX…”

**Responses from the authors:** *Per suggestion, it has been changed according.*

**Conclusion**

COL3A1 is in italic, but the other genes are not, please follow a pattern throughout the text. I suggest reporting mRNAS in italic.

**Responses from the authors:** *Thanks for your excellent comment.A pattern has been followed ithroughout the text.According to your suggestion, use italics for all genes and mRNA*

**Supplementary material:**

Table S1, S2 and S3: Unnecessary to report. I suggest to remove.

**Responses from the authors:** *Thanks for this comment. They are removed.*

Figure S5 should be cited in the text

**Responses from the authors:** *Thanks for your excellent suggestions.Reference to Figure S5 has been added to the article(L95).*

Reviewer #2:

I got your point and the comparative dct method seems correct, but I still have some serious concerns about the statistics you should have done after that.At the Data processing and statistical topic, 2-△△Ct is a method to access the fold relative gene expression, which means that using this mathematical model you are able to compare different groups in terms of gene expression level e.g. how many times a gene is more expressed in one group than another. In another hand, in order to access if there is a statistical difference between groups you must use a proper statistical model/test (parametric or non-parametric) after ddct method; and I still have not seen it in the text. A one-way ANOVA or t-test would be enough for you. Once the statistical metrics have not been properly addressed I can not trust any qPCR results.

I strongly suggest you to perform the statistical test and potential corrections and resubmit the manuscript.

**Responses from the authors:** *We are very sorry that our misunderstanding did not effectively solve the problems raised by the reviewer, which increased your workload. Your patient explanation gives us a correct understanding.Thanks for your excellent summary comments. Your points are well taken. Per your suggestion,We have modified and supplemented this part.*

*After comparing the genes expression levels of different groups with 2-△△Ct method.The data from all experiments were first tested for collation and outliers. Statistical analysis was performed using SPSS software, version 20.0 (IBM-SPSS, Chicago, Illinois, USA).One-way ANOVA and Duncan multiple comparisons test were used to evaluate the differences expression of different genes in different varieties. The data were presented as mean±standard deviation (SD).*

*We have carefully conducted statistical tests and potential corrections.We are looking forward to your "acceptance" suggestions and responses！* *If there is anything else we need to do, please feel free to let us know.*
